# Analytical Study on Current Trends in the Clinico-Mycological Profile among Patients with Superficial Mycoses

**DOI:** 10.3390/jcm12093051

**Published:** 2023-04-22

**Authors:** Shreekant Tiwari, Monalisah Nanda, Swetalona Pattanaik, Ganiga Channaiah Shivakumar, Bukanakere Sangappa Sunila, Marco Cicciù, Giuseppe Minervini

**Affiliations:** 1 Department of Microbiology, Hi-Tech Medical College and Hospital, Bhubaneswar 751025, India; drshreekant@rediffmail.com (S.T.); drsweta06@gmail.com (S.P.); 2 Department of Dermatology, Shri Jagannath Medical College and Hospital, Puri 752002, India; drmonalisah@yahoo.com; 3 Department of Oral Medicine and Radiology, People’s College of Dental Sciences and Research Centre, People’s University, Bhopal 462037, India; 4 Department of Prosthodontics and Crown & Bridge, JSS Dental College and Hospital, JSS Academy of Higher Education and Research, Mysuru 570015, India; drsunilasangappa@gmail.com; 5 Department of Biomedical and Surgical and Biomedical Sciences, Catania University, 95123 Catania, Italy; 6 Multidisciplinary Department of Medical-Surgical and Dental Specialties, University of Campania, Luigi Vanvitelli, 80138 Naples, Italy

**Keywords:** antifungal therapy, dermatology, dermatophytes, systematic mycoses

## Abstract

Infections affecting the superficial keratinized layer of the skin, nails, and hair are referred to as dermatophytosis and dermatomycoses, which constitute the most common type of fungal infection that affects people. This clinical ailment has a prevalence of between 30 and 60% and is more common in India’s hot, muggy, tropical climate. Examining the prevalence of superficial mycoses (SM), their clinical symptoms, and the fungal species that were identified as the disease-causing agents were the main objectives of the current study. This study comprised 250 clinically confirmed patients with SM who visited our dermatology department over the course of a year. Skin scrapings, nail clippings, and hair samples were gathered, mounted, and cultured using KOH. Macroscopic examination of culture, tease mount, and phenotypic tests were used to identify the species. The age group of 11–20 years (29%) had the highest prevalence of SM out of the 250 clinically verified cases of the condition that were included in our study, followed by 21–30 years (20%) and 31–40 years (18%). *Candida albicans*, dermatophytes, and non-dermatophytic moulds were the three most prevalent fungal isolates. The most typical dermatophyte isolate was *T. rubrum*, which was primarily found in *Tinea corporis* (TCo), *Tinea cruris* (TCr), and *Tinea faciei* (TFa). *T. mentagrophytes* was the second most frequent isolate. According to our investigation, it was determined that non-dermatophytic moulds constitute a significant contributor to the development of SM in addition to dermatophytes.

## 1. Introduction

Skin, hair, and nail fungal infections known as SM are brought on by dermatophytes, yeasts, and non-dermatophyte moulds [1]. According to the clinical kinds, different agents are the causal parties. *Malassezia furfur*, a common yeast found on the skin, is the culprit behind *Pityriasis versicolor*. It is common to refer to dermatophytosis as “Tinea” [2,3]. Since it is the first and most prevalent kind of SM, it is widely investigated [3]. It is brought on by a diverse collection of fungi known as dermatophytes, some of which have taken up residence in humans and are referred to be anthropophilic.

Multiple investigations [4,5,6,7,8,9] on SM have been carried out in India. Our patients are from Eastern Odisha, a region of India where the relative humidity and temperature are high for most of the year and the monsoons are intense. Due to the high levels of environmental dampness in this region, sweat evaporation is slowed, which encourages fungal development and a high prevalence of fungi-related disorders. Mentioned in Table 1 are the findings of a similar study on the incidence of SM in the eastern Odisha region [3].

The second most frequent kind of SM typically observed in adults is tinea versicolor. Patchy skin discolouration, primarily on the chest, neck, and back, is what it is known for. The third type of onychomycoses resembles *Tinea unguim*. The majority of these infections are asymptomatic, and patients typically seek medical counsel for aesthetic or mild pruritic reasons. Before beginning antifungal therapy, a laboratory diagnosis is crucial since some diseases require long-term antifungal therapy while others do not. To treat SM, numerous antifungal medications are used. The susceptibility pattern in dermatophytes of the same species might occasionally vary. Infections with fungi that are superficial to the skin are the most typical fungi infections to occur worldwide [10,11,12,13,14]. The World Health Organization (WHO) estimates that 20–25% of people globally have a superficial mycotic infection. [15] Various nations have different levels of prevalence. Dermatophytes are by far the most common and significant cause of cases due to their widespread involvement in the general population and their global frequency. [16] Dermatophytic fungi infect roughly 20–25% of the world’s population, and the prevalence is steadily rising. Any topographical region can have dermatophytoses; no human race is immune in coverage and prevalence. The types of dermatophytes that exist vary geographically and throughout time, depending on factors including personal hygiene, the environment, and the sensitivity of the individual. [17] The differences in the dermatophytosis distribution pattern are ascribed to social practices, labour migration, troop movements, immigration, and frequent international travel. [18] Ringworm, also known as dermatophytosis, is a fairly prevalent superficial fungal skin condition. In tropical and subtropical areas such as India, where temperatures and humidity are high for most of the year, it is more prevalent.

The goal of the current investigation was to determine the prevalence of SM, their clinical manifestation, and the species of fungi isolated as the disease-causing agents.

## 2. Materials and Methods

### 2.1. Study Hypotheses

Using this study, we aimed to investigate the various microbial species responsible for causing SM in our study subjects who presented to us in our dermatology department in the region of Eastern Odisha.

### 2.2. Study Design

Ours was an observational study, the primary objective of which was to determine the occurrence of SM, their clinical manifestation, and identify the fungi isolated as the disease-causing agents.

### 2.3. Sample Size and Sampling

This study comprised 250 cases of SM with a clinical diagnosis, lasting for a period of one year in Eastern Odisha. A thorough clinical history was taken, including information on the patient’s age, sex, type of lesions, duration, occupation, and similar complaints in the family. The Institutional Ethics Committee at Hi-Tech Medical College and Hospital, Bhubaneshwar, Odisha, India, authorized the protocol, which was carried out in accordance with the Helsinki Declaration (Protocol number: “ HMCH/IEC/2021/156”, date: 11 September 2021). Written informed consent for involvement was provided by each patient and their guardians. Informed consent was obtained from the parent prior to data collection.

Prior to choosing the sample, proper labelling of the specimen in a sterile container was also performed.

### 2.4. Inclusion/Exclusion Criteria

We did not restrict the selection criteria for the samples we used in our study to factors such as age, gender, demographics, or the patient’s overall health or disease.

The domains of our investigation did not include patients who had recently undergone major surgery, who had recently been discharged or hospitalized in the ICU, or both. Patients who required a ventilator or were too weak were also excluded from the sampling, as were people who had had any kind of antifungal or antibiotic therapy 2–3 months prior to the start of our study.

Furthermore, we did not include immunocompromised patients and HIV-positive individuals in our study since their inclusion could have introduced potential confounding factors and made it difficult to draw clear conclusions about the trends in these populations. Moreover, several of the risks associated with their participation in the study outweighed the potential benefits (which were primarily ethical concerns), and as a result, these populations were excluded.

### 2.5. Laboratory Procedures

Prior to sample collection, each case received a medical, hygienic, socioeconomic, and habitual profile. After thoroughly cleaning the affected area with 70% ethanol, skin scales and crusts from the edges of inflamed lesions, nail clippings with subungual scrapings, and epilated lustreless affected hair with roots were collected in a dark background paper envelope for easy visualisation and left overnight for absorption of moisture. The following day, direct microscopy was used to examine nail clippings that had been 40% KOH-treated the night before. Then, 10% KOH was used to examine skin and hair samples up close under the microscope. Two more clinical sample sets were inoculated into two sets of Sabouraud’s Dextrose agar tubes, one with 0.05% chloramphenicol and the other with 0.05% chloramphenicol and 0.5% cycloheximide. One clinical sample set was used for direct microscopy. Growth was monitored twice weekly for at least three weeks while these tubes were cultured at 25 °C and 37 °C before being labelled as negative. Any diffusible pigment as well as colony characteristics including texture, surface, and colour on both the obverse and reverse were identified as the growth of fungal isolates. In the bulk of the cases, the fungal isolate was identified by directly examining the mount from SDA tube growths with lactophenol cotton blue.

For *C. albicans* and the other fungal specimens, we used a sugar assimilation test, where different types of sugars, such as glucose, lactose, maltose, and sucrose, are added to a culture of the yeast or fungus being tested [18]. The organism’s ability to metabolize or “assimilate” these sugars was then observed over time. This test is commonly used to identify different species of yeasts and fungi, including Candida species. In the presence of glucose, *C. albicans* produced lactic acid, which was detected using phenol red, resulting in a drop in the pH of the medium. Based on the pattern of sugar assimilation, the yeast or fungus can be identified to the species level.

### 2.6. Statistical Analysis

We created the numbers needed for our study’s statistical analysis using SPSS (Statistical Package for the Social Sciences, Chicago, IL, USA) Version 26.0.

## 3. Results

The age range of 11–20 years (29%) had the highest prevalence of SM out of the 250 clinically confirmed cases of the condition that were included in our study, followed by 21–30 years (20%) and 31–40 years (18%).

Out of the 250 participants in our study, there were 130 males and 120 females. Table 2 clearly displays these numbers.

**Figure 1:**
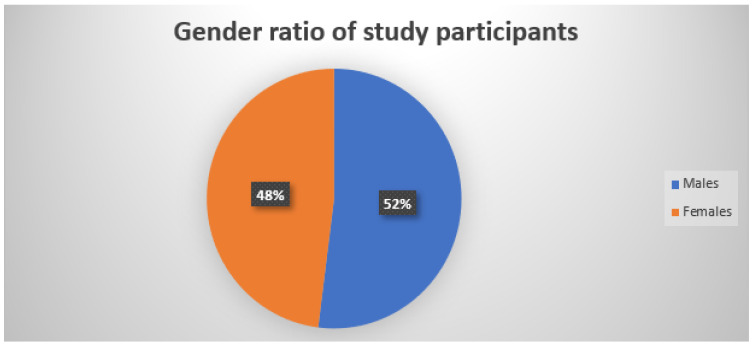
Pie chart representing the gender ratio of subjects enrolled in this study.

**Figure 2:**
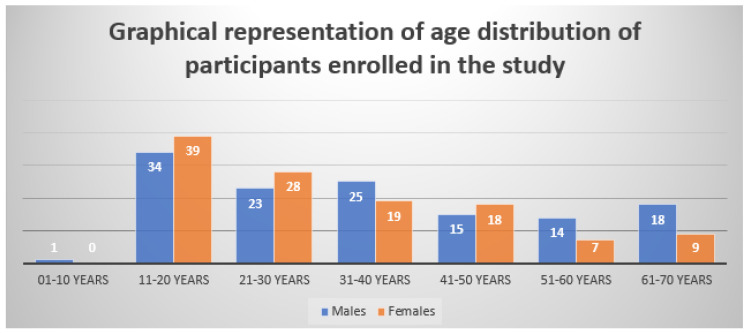
Graphical representation showing the age distribution of participants enrolled in this study according to the details mentioned in Table 2.

**Figure 3:**
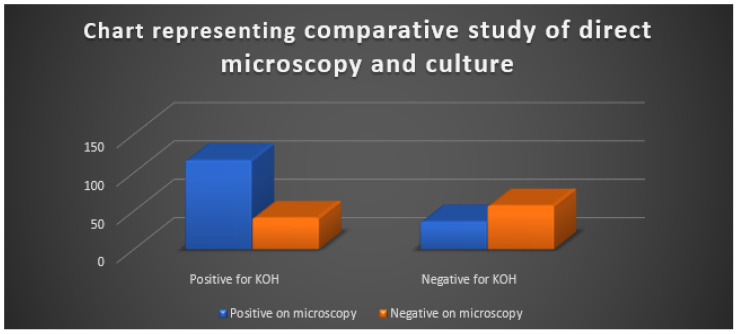
Chart representing the comparative study of direct microscopy and culture.

Figure 1 shows a pie chart representing the gender ratio of subjects enrolled in the study as mentioned in Table 1. Figure 2 displays the age ranges of all the participants enrolled in the study, and Figure 3 shows a representation of the comparative study involving the results obtained using the culture and microscopy respectively.

A total of 116 cases (about 46%) out of the 250 cases had positive results from both microscopy and culture. Overall, 36 instances (14%) had both positive cultures and negative direct microscopy results, while 41 cases (16%) were only positive with direct microscopy, the details of which are mentioned in Table 3 given below as well as visually represented in Figure 3. The analysis indicates that out of the total 250 cases studied, 116 cases (about 46%) showed positive results for superficial mycoses when both microscopy and culture tests were performed. This suggests that the combination of these two tests may be the most reliable method for diagnosing superficial mycoses. Further analysis reveals that in 36 cases (14%), the culture test was positive, but the direct microscopy test was negative. This could be due to a variety of factors, such as the presence of non-viable fungal elements or the inability of the microscopy technique to detect certain types of fungal elements. In 41 cases (16%), only the direct microscopy test was positive, while the culture test was negative.

Overall, the results of this study highlight the importance of using both microscopy and culture tests to diagnose superficial mycoses accurately. However, the limitations of these tests must also be considered, and further research may be needed to develop more sensitive and specific diagnostic methods.

As shown in Table 4, a total of 133 different microbial species were isolated from the study population. Of these, dermatophytes were the most prevalent superficial fungal species, accounting for 97 cases (73%), non-dermatomycotic moulds for 27 cases (20%), and candidiasis for the remaining 7 (7%).

Table 5 illustrates the relationship between etiological agents and clinical categories. Dermatophytes, *C. albicans*, and non-dermatophytic moulds were the three most prevalent fungal isolates. The most prevalent dermatophyte isolate was *T. rubrum*, which was mostly found in TCo, TCr, and TFa. *T. mentagrophytes*, which was most frequently isolated from TCo and TCr, was the second most typical isolate. The fungus that was responsible for onychomycosis was isolated. The majority of non-dermatophytic moulds (NDMs) were found to be isolated from nail infections in individuals who had a history of trauma.

## 4. Discussion

In our country, SM is particularly common since the hot, humid weather is ideal for the development of infection. Since long-term treatment may be necessary in some cases, it is important to identify the fungus that is causing the SM both for epidemiology and for therapy. The key factor contributing to the frequency of this disorder in the population is that most people are usually ignorant of the illness, fail to treat the early lesions, and present with lesions at several sites as a result.

In our study, SM was more common in men than in women and was more common in those between the ages of 11 and 20 (29%). These results are comparable to those of additional studies conducted by Sharma et al. [19]. Males (48.04%) were more frequently impacted than females (51.96%), according to Madhuri et al. [20], whereas Karmarkar et al. [21] found that the most common age range affected was 0 to 10 years (28.4%). Because they participate in more physically demanding outdoor activities and perspire more, young boys may have a higher incidence.

According to Patel et al. [22] and Patwardhan et al. [23], TCo was the most common clinical type seen in adult males, followed by TCr and TFa. The higher prevalence of the condition in men may be attributed to increased perspiration in the groin region, decreased ventilation due to tight-fitting clothing, and greater susceptibility to dermatophyte infections in males.

According to Madhuri et al. [20] and Patwardhan et al. [23] as well as other studies, females were more likely to have *Tinea pedis* and onychomycosis. *Pityriasis versicolor* was found in 8.8% of cases due to the lipophilic characteristic of *Malassezia* species, which is crucial for their growth; individuals between the ages of 21 and 30 were most frequently impacted. However, the years with the highest sebum production are usually the ones when the yeasts are present. These findings are on par with past research by Das et al. [16] and Nazish et al. [24].

The KOH preparation used in our experiment showed good sensitivity when compared to the culture. These findings are comparable to Karmakar et al.’s prior research findings. Bindu et al. [25] and Singh et al. reported KOH sensitivity [26]. Twenty instances had favourable microscopy results but negative results from cultures. This variance may be brought on by the nonviability of fungal components in some circumstances and the inadequate sample brought on by the extremely small lesions.

Using microscopy, 36 (14%) were negative but cultured positively. These alterations result from poor information and incorrect diagnoses since short fungal components were present.

*T. rubrum*, *T. mentagrophytes*, and *E. flocosum* isolates were the most frequently found fungi in most clinical kinds. The findings of this study are comparable with Sen et al. [27] and Patwardhan et al. [23]. Seven instances in our investigation were caused by *T. tonsurans*. From eight cases of TCo and one case of TFa, *T. verrucosum* was isolated. Mathur et al. [28] and Sahai et al. [29] have both reported on the high occurrences of *T. verrucosum*. Only eight incidences (isolation of *T. interdigitale*) were reported.

Other studies did not reveal any evidence of *T. interdigitale* infection. In our investigation, the majority of *Candida albicans* and non-dermatophytes were identified from onychomycosis. NDM contained *Aspergillus niger* species and species of *Aspergillus flavus*, *Penicillium*, and *Fusarium*. These results align with those of Madhuri et al. [11]. Only NDM isolated repeatedly in pure culture together with a positive KOH result were regarded as noteworthy.

Both Verma et al. [30] and Bhat et al. [31] conducted studies on SM in different regions of India. Verma et al. [30] analysed clinical records and histopathological features of SM cases in northeast India. Bhat et al. [31] evaluated different types of SM in Karnataka, India. Verma et al. [30] analysed 70 patients with SM, of whom 44 were males and 26 were females. The majority of patients were aged between 20 and 60 years, and 87% were from rural areas. Agricultural workers accounted for 92% of the patients. The lower limb was afflicted more in comparison to the upper limb in the study. This is unlike our findings, where we could not consider the various regions of the body in the study participants. Chromoblastomycosis was the most common SM seen in northeast India. Bhat et al. [31] analysed 25 patients with SM, of whom 16 had chromoblastomycosis, 4 had mycetoma, 4 had sporotrichosis, and 1 had rhinoentomophthoromycosis. Both studies found that chromoblastomycosis was the most common SM in their respective regions of India [30,31]. The lower limb was the most commonly affected site in both studies, and the majority of patients were agricultural workers. Verma et al. found that fungal culture was positive in only 55.7% of cases, while Bhat et al. 2015 did not report the culture positivity rate. In terms of the similarities observed in the findings, both studies highlight the importance of a detailed clinical history, histopathological examination, and culture for the accurate diagnosis of SM [30,31]. Verma et al. [30] found that the most common site of involvement was the lower limb followed by the upper limb, while Bhat et al. [31] reported that the extremities were the most commonly affected site, with the lower limb being the most affected. Both studies also found that agricultural workers were at higher risk of developing SM, with a history of trauma being obtained from a significant proportion of patients. Verma et al. [30] also found a higher prevalence of chromoblastomycosis compared to sporotrichosis, while Bhat et al. [31] found an equal prevalence of both. Histopathology and clinical presentation were important tools for diagnosis in both studies, and most patients responded well to therapy.

A review conducted by Chakravarti et al. [32] in 2008 discusses the increase in fungal infections in immunocompromised patients in tertiary care centres in India. However, there was limited data on the burden of opportunistic mycoses in India at that time. The emerging trends showed an increased incidence of invasive candidiasis, aspergillosis, and zygomycosis. In addition, unique cases of fungal rhinosinusitis, penicilliosis marneffei, and zygomycosis due to *Apophysomyces elegans* were reported. Invasive candidiasis was the most common opportunistic mycosis, with *Candida tropicalis* being more prevalent than *C. glabrata* or *C. parapsilosis*. Invasive aspergillosis was the second most common and was expected to have a high prevalence in Indian hospitals due to ongoing construction activities without proper barriers. Invasive zygomycosis was also reported to be a concern, as India reported the highest number of cases worldwide, commonly observed in patients with uncontrolled diabetes mellitus. The article concluded that there was a need for accurate diagnostic laboratories, rapid diagnosis, and refinement in antifungal strategies in India. Another review conducted by Agarwal et al. [33] provides a detailed overview of chromoblastomycosis, a chronic fungal infection affecting the skin and subcutaneous tissues, and its prevalence in India. The authors reviewed 169 cases published in the English literature from India and found a significant increase in the reported cases since 2012, with a majority of patients involved in agricultural activities, and the lower extremity was the most common site of infection. *Fonsecaea pedrosoi* was identified as the most common fungal pathogen, and itraconazole and terbinafine were the most commonly used antifungals with variable outcomes. The study highlights the need for increased awareness and early clinical suspicion of the disease, as well as the development of more effective treatment strategies. Overall, the abstract provides useful insights into the epidemiology, clinical presentation, and management of this fungal infection. Opportunistic fungal infections are a significant public health problem globally, particularly in Latin America. In this region, certain mycoses, such as candidiasis, cryptococcosis, trichosporonosis, aspergillosis, and fusariosis, are particularly prevalent. The epidemiology of these mycoses in Latin America differs from that in other parts of the world, and a review by Nucci et al. [34] provided valuable insights into the epidemiologic features of these diseases. One of the most notable findings in the review was that the incidence of candidemia in Latin America was markedly higher than that reported in North America and Europe. This was a significant observation that highlights the importance of candidemia as a public health problem in Latin America. The review also highlighted the species distribution of *Candida* causing bloodstream infections in Latin America, with *Candida parapsilosis* or *Candida* tropicalis being more common than *Candida glabrata*. This finding was in contrast to North America and Europe, where *Candida glabrata* was the most commonly isolated non-albicans *Candida* species causing candidemia. This difference in species distribution, the authors believed, could have implications for antifungal therapy, as different *Candida* species might have different susceptibilities to antifungal drugs. Furthermore, the review noted that the epidemiologic features of other mycoses in Latin America, such as cryptococcosis, trichosporonosis, aspergillosis, and fusariosis, differed from those in other regions. For example, cryptococcosis was more common in Latin America than in North America or Europe, and the incidence of trichosporonosis was higher in Latin America than in Asia or Europe. These observations highlighted the importance of considering regional differences in the epidemiology of fungal infections when developing treatment and prevention strategies. The review also identified various risk factors for opportunistic fungal infections in Latin America, including HIV infection, hematologic malignancies, solid organ and hematopoietic stem cell transplantation, and the use of broad-spectrum antibiotics and immunosuppressive agents. These risk factors were consistent with those identified in other regions, but their relative importance might have differed in Latin America due to regional differences in the incidence and prevalence of these risk factors.

*Penicillium* and other similar fungi are often considered lab contaminants because they are commonly found in laboratory settings and can contaminate samples, leading to false results [34]. However, there are certain instances where these fungi are actually the cause of disease, such as in cases of onychomycosis, a fungal infection of the nails. When identifying *Penicillium* or similar fungi as the cause of onychomycosis, it is important to follow the Welsh criteria. The Welsh criteria are a set of guidelines used to determine whether a fungal infection is truly the cause of a patient’s symptoms or if it is simply a contaminant from the lab [34].

One of the main factors contributing to the change in dermatophyte epidemiology is the way people dress [34]. In many parts of the world, traditional clothing has given way to more Western-style clothing, which often includes tight-fitting garments made from synthetic materials [34]. These types of clothes trap moisture and heat, creating a perfect environment for dermatophytes to grow [34]. As a result, dermatophyte infections have become more common in areas where Western-style clothing is prevalent. Another factor contributing to the change in dermatophyte epidemiology is lifestyle changes [34]. As people have become more sedentary and spend more time indoors, they are more likely to develop dermatophyte infections [34]. This is because dermatophytes thrive in warm, humid environments, such as gyms, locker rooms, and swimming pools. Additionally, poor hygiene practices can also contribute to the spread of dermatophyte infections [34]. Steroid abuse is another factor that has contributed to the changing epidemiology of dermatophytes [34]. Steroids are often used to treat a variety of medical conditions, but they can also have the unintended consequence of weakening the immune system [34]. This can make individuals more susceptible to dermatophyte infections, which can be difficult to treat in individuals with compromised immune systems [34]. Overall, the changing epidemiology of dermatophytes is a complex issue that is influenced by a variety of factors, including changes in clothing, lifestyle, and steroid abuse. Understanding these factors is important for developing effective strategies for the prevention and treatment of dermatophyte infections. To prevent dermatophyte infections, it is important to practice good hygiene, avoid tight-fitting clothing made from synthetic materials, and seek medical attention promptly if an infection is suspected.

Tinea capitis, commonly known as scalp ringworm, is a fungal infection of the scalp caused by dermatophytes [34]. It primarily affects children, although it can occur in adults as well. The most common causative agents of tinea capitis are Trichophyton tonsurans and Microsporum canis [34]. Symptoms of tinea capitis may include scaly, itchy, or inflamed patches on the scalp, hair loss, and broken hairs at the surface of the scalp. The infection can also spread to other areas of the body, such as the face or neck [34]. Diagnosis of tinea capitis is typically made using a combination of clinical examination, microscopy, and culture. Treatment for tinea capitis usually involves either topical or oral antifungal medications [34]. Oral medications may be necessary for more severe cases or for infections caused by certain types of dermatophytes. In addition to medication, hair hygiene is an important part of managing tinea capitis, as the fungus can survive in hair and scalp debris [34]. Prevention of tinea capitis involves good hygiene practices, such as washing hair regularly with mild shampoo and avoiding sharing combs, brushes, or hats with others [34]. Education about the infection and its spread is also important, particularly in schools and other communal settings where children are likely to come into contact with each other’s hair and scalp [34].

Our study has revealed that SM is a common problem in Eastern Odisha, with a high prevalence of fungal infections among patients seeking dermatological care, and identified the most common types of SM in this population. The findings obtained also highlight the importance of accurate diagnosis and appropriate treatment for SM, as misdiagnosis and inappropriate use of antifungal agents can lead to the emergence of drug-resistant fungal strains. All in all, this investigation provides valuable information on the prevalence and clinical characteristics of SM in Eastern Odisha and highlights the need for effective diagnosis and management of fungal infections in this part of the world. This study also underscores the need for awareness among healthcare providers and the public regarding the prevalence and clinical characteristics of SM in Eastern Odisha.

Some limitations were identified in our investigation that may affect the accuracy and generalizability of the results. These limitations include issues related to sampling, diagnostic methods, and data analysis. One limitation of this study is the sampling method. This study was conducted on a sample of 250 clinically confirmed cases of SM who visited the dermatology department over the course of a year. This sampling method may not be representative of the broader population, as individuals who seek medical care for skin conditions may differ in important ways from those who do not. Additionally, this study did not include individuals who may have had SM but did not seek medical attention, which may underestimate the true prevalence of the condition. Another limitation of this study is the diagnostic method used to identify fungal species. The authors state that skin scrapings, nail clippings, and hair samples were gathered and cultured using KOH. While KOH is a common and inexpensive diagnostic method for fungal infections, it has limitations in terms of sensitivity and specificity. Other diagnostic methods, such as fungal cultures or PCR, may be more sensitive and accurate in identifying fungal species. Different fungal species can cause a range of clinical symptoms, and the absence of this information (on the clinical symptoms of the patients) in the sample makes it difficult to draw conclusions about the specific types of SM that are most prevalent in the study population. Finally, we were unable not provide information on the socioeconomic status or other demographic characteristics of the sample, which may be relevant to understanding the distribution and prevalence of SM. Certain risk factors, such as poor hygiene or crowded living conditions, may be more prevalent in certain demographic groups and may contribute to the development of fungal infections.

## 5. Conclusions

Our study on the current trends in the clinico-mycological profile among patients with superficial mycoses in Eastern Odisha, a hot and humid part of the country, revealed that the age range of 11–20 years had the highest prevalence of SM out of the 250 clinically confirmed cases of the condition included. Furthermore, we found that dermatophytes were the most prevalent superficial fungal species, accounting for 73% of the total cases. Our analysis also showed that the combination of both microscopy and culture tests is the most reliable method for diagnosing superficial mycoses accurately. However, the limitations of these tests must also be considered, and further research may be needed to develop more sensitive and specific diagnostic methods. These findings have several future implications. Firstly, healthcare providers in Eastern Odisha should be aware of the high prevalence of superficial mycoses in the younger population and take necessary measures to prevent its spread. Secondly, given the predominance of dermatophytes in our study, efforts should be made to develop effective treatment strategies for this fungal species. Lastly, future research can focus on developing more sensitive and specific diagnostic methods for superficial mycoses to improve patient outcomes.

## Figures and Tables

**Table 1:** Abbreviations used in the study.

Type of SM Identified	Number of Cases Reported
Tinea corporis	590
Tinea corporis and tinea cruris	226
Tinea cruris	193
Tinea faciei	82
Tinea cruris + tinea faciei + tinea manuum	65
Tinea pedis	22
Tinea manuum	13
Tinea incognito	6
Tinea barbae	3

**Table 2:** Age and gender characteristics of the individuals selected for this study.

Age	Males	Females	Total	Percentage
**01–10 years**	1	0	1	0%
**11–20 years**	34	39	73	29%
**21–30 years**	23	28	51	20%
**31–40 years**	25	19	44	18%
**41–50 years**	15	18	33	13%
**51–60 years**	14	7	21	8%
**61–70 years**	18	9	27	11%

**Table 3:** Analysis of culture using microscopy.

Culture	Positive for KOH	Negative for KOH	Total
**Positive on microscopy**	116 (46%)	36 (14%)	152 (61%)
**Negative on microscopy**	41 (16%)	57 (23%)	98 (39%)
**Total**	157 (63%)	93 (37%)	250 (100%)

**Table 4:** Percentages of microbial species isolated from the study sample.

*Microbial Species Isolated*	*Number*	*p-Value*
**Dermatophytes**	97 (73%)	<0.05
**Non-dermatophyte moulds**	27 (20%)	<0.05
**Candida albicans**	7 (7%)	<0.01
**Total**	133 (100%)	
***Microbial species isolated***	*Number*	*p-Value*
**Dermatophytes**	97 (73%)	<0.05
**Non-dermatophyte moulds**	27 (20%)	<0.05
**Candida albicans**	7 (7%)	<0.01

**Table 5:** The prevalence of fungal isolates and their relation to their clinical type.

Species Isolated	Dermatophytes						Non-Dermatophytic Moulds				*Candida albicans*
	*E. floccosum*	*T. interdigitale*	*T. mentagrophytes*	*T. rubrum*	*T. tonsurans*	*T. verrucosum*	*A. flavus*	*A. niger*	*Penicillium* spp.	*Fusarium* spp.	
***T. corporis***	9	7	15	21	7	8	-	2	-	3	-
***T. cruris***	-	-	7	12	-	1	-	-	3	-	-
***T. pedis***	2	-	-	-	-	-	-	-	-	-	-
***T. manuum***	-	1	-	2	-	-	-	-	2	-	-
***T. faciei***	1	-	3	-	-	1	-	-	-	-	-
***Onychomycosis***	-	-	-	-	-	-	4	4	3	6	7

## Data Availability

The data presented in this study are available on request from the corresponding author. The data are not publicly available due to concerns pertaining to privacy and identity of the consenting participants that were involved in this study.

## Abbreviations

The following abbreviations are used in this manuscript:

SM	Superficial mycoses
TCo	Tinea cruris
TCr	Tinea corporis
TFa	Tinea faciei
KOH	Potassium hydroxide
NDM	Non-dermatophytes
*T. rubrum*	*Trichophyton rubrum*
*T. mentagrophytes*	*Trichophyton mentagrophytes*
*E. floccosum*	*Epidermophyton floccosum*
*T. tonsurans*	*Trichophyton tonsurans*
*T. verrucosum*	*Trichophyton verrucosum*
*T. interdigitale*	*Trichophyton interdigitale*
*C. albicans*	*Candida albicans*
KOH	Potassium hydroxide
